# The Histamine-Associated Inflammatory Landscape of Endometriosis: Molecular Profiling of HDC, HRH1-HRH4, and Cytokines Across Lesion Subtypes

**DOI:** 10.3390/ijms27010212

**Published:** 2025-12-24

**Authors:** Renata Voltolini Velho, Julia Hannah Freitag, Arie Maeve Brueckner, Laura Thalmeier, Jonathan Pohl, Sylvia Mechsner

**Affiliations:** 1 Department of Gynecology Charité with Centre of Oncological Surgery, Endometriosis Research Centre Charité, Charité University Hospital, Campus Virchow-Klinikum, Augustenburger Platz 1, 13353 Berlin, Germany; renata.voltolini-velho@charite.de (R.V.V.); julia-hannah.freitag@charite.de (J.H.F.); arie.brueckner@gmail.com (A.M.B.); laurathalmeier@t-online.de (L.T.); 2 Institute for Pathology, Charité University Hospital, Campus Mitte, Charitéplatz 1, 10117 Berlin, Germany; jonathan.pohl@charite.de

**Keywords:** endometriosis, histamine, histamine receptors, neuroimmune signaling, mast cells, nociception, inflammation

## Abstract

Pain in endometriosis involves not only nociceptive but also neuropathic and neurogenic components, reflecting its complex nature. Histamine, a biogenic amine, has emerged as a critical mediator connecting inflammation and nerve sensitization. This study aimed to characterize histamine receptor (HRH1–HRH4) expression, localization, and related inflammatory mediators in peritoneal, deep infiltrating, and ovarian endometriosis. Gene expression datasets were analyzed, and immunofluorescence staining of endometriotic lesions was performed using immune and neuronal markers. Histamine and its metabolite methylhistamine were quantified in serum, peritoneal fluid, and urine samples. *HDC* expression was significantly elevated in all endometriotic lesions compared with controls (all *p* < 0.01), paralleling increased *IL-6*, *COX-2*, *NGF*, and *NGFR* levels (*p* < 0.0001). In contrast, *HRH1–HRH4* transcript levels showed no significant differences between groups. Immunofluorescence demonstrated robust HRH1–HRH4 protein expression in epithelial, immune, and nerve fibers, with subtype-specific colocalization patterns. Serum histamine concentrations were significantly higher in endometriosis patients than controls (0.484 vs. 0.153 ng/mg protein; *p* = 0.0014), whereas peritoneal histamine and urinary methylhistamine showed no group differences. Overall, these findings highlight histamine signaling as a potentially important component of endometriosis pathophysiology and point toward new directions for mechanistic studies and therapeutic exploration.

## 1. Introduction

Endometriosis is a chronic, estrogen-dependent inflammatory condition that affects 7–10% of menstruating individuals worldwide [1,2]. While most commonly diagnosed in individuals of reproductive age, endometriosis has also been reported in postmenopausal and transgender patients, including those who have undergone hysterectomy, highlighting its complex and persistent nature [3,4]. This chronic condition involves the development of lesions containing endometrial-like tissue and smooth muscle cells outside of the uterus, typically on the ovaries, fallopian tubes, and peritoneum. These lesions can lead to a variety of symptoms, including dysmenorrhea, both cyclic and acyclic lower abdominal pain, dysuria, dyspareunia, and infertility [1,5,6].

Despite ongoing research, the etiology of endometriosis-associated pain remains only partially understood. Increasing evidence implicates neuroinflammatory mechanisms in its pathophysiology [7,8]. A key feature observed in endometriosis lesions is the increased density of sensory nerve fibers in proximity to immune cells such as mast cells [9,10,11]. Among the mediators released by activated mast cells, histamine has garnered increasing attention due to its pro-inflammatory and nociceptive properties [12,13].

Histamine, a biogenic amine synthesized from the amino acid L-histidine by histidine decarboxylase (HDC), is a pleiotropic factor with a dichotomous nature, acting as both a potent inflammatory mediator and an integral component of homeostatic functions [14]. Examples of its latter behavior include its role in the sleep–wake cycle, the regulation of body temperature and blood pressure, and the secretion of gastric acid. While its inflammatory roles include the recruitment and activation of immune cells (Mast cells, eosinophils, and NK cells), the stimulation of pro-inflammatory cytokine production (IL-6 and TNF-alpha), and the dysregulation of blood vessel formation, enabling immune cell infiltration [14,15]. Crucially, histamine is also an integral mediator in the processing of nociceptive information, both in an antinociceptive manner in the central nervous system (CNS) and in a nociceptive manner in the peripheral nervous system (PNS) [16].

Histamine acts via four distinct G-protein-coupled receptors, HRH1, HRH2, HRH3, and HRH4, which differ in localization, binding affinity, and signaling cascades. HRH1 is expressed in various cell types, including muscle, nerve, and endothelial cells, as well as immune cells such as neutrophils, eosinophils, and mast cells. HRH1 plays a significant role in allergic inflammation, driving cellular migration, nociception, vasodilation, and bronchoconstriction [17]. HRH2 is predominantly expressed in the gastrointestinal tract, such as on parietal cells in the stomach, where it regulates the role of gastric acid secretion. HRH3 is primarily expressed in the CNS, where it acts as both an auto- and heteroreceptor modulating the release of other neurotransmitters and cognitive processes. HRH4 is primarily found on immune cells, such as resident mast cells in the skin, and is involved in immune responses and chemotaxis. HRH4-mediated mast cell activation is reported to regulate a mighty inflammatory cascade triggering the release of inflammatory mediators and the migration of inflammatory cells to the site of inflammation [15,18]. HRH1-2 are low-affinity receptors, whilst HRH3-4 are high-affinity receptors, activating at low levels of histamine [19,20,21].

The involvement of histamine in endometriosis is supported by studies reporting increased numbers of degranulating mast cells within lesions and the proximity of tryptase-positive mast cells to nerve fibers, implicating neuroimmune interactions in the generation of pain [11,22]. Additionally, histamine is known to stimulate nerve growth factor (NGF) expression, which, in turn, enhances the synthesis of neuropeptides such as substance P (SP) and calcitonin gene-related peptide (CGRP)—critical modulators of pain transmission [23,24]. Animal studies further underscore histamine’s role in pain sensitization. Zhu et al. [25] demonstrated in a rat model of surgically induced endometriosis that oral administration of ketotifen (a mast-cell stabilizer and HRH1 antagonist) significantly suppressed the development of hyperalgesia, likely by reducing mast-cell activity within lesions and thereby dampening peripheral sensitization. Additional studies across diverse pain models have shown that mast-cell stabilizers and histamine-receptor antagonists reduce hyperalgesia [25,26], pelvic pain [27], inflammatory pain [28], and postoperative nociception [29], further supporting a central role for histamine-driven pathways in endometriosis-associated pain.; for instance, ketotifen, a mast cell stabilizer and HRH1 antagonist, has been shown to alleviate hyperalgesia in a rat model of surgically induced endometriosis.

Despite growing evidence that histamine plays a significant role in neuroimmune signaling, its specific role in endometriosis-related pain remains poorly understood. This study aims to fill this gap by examining histamine receptor expression and localization in endometriotic tissue, assessing histamine-related inflammatory mediators, and measuring histamine and its metabolites in urine, peritoneal fluid, and serum. We hypothesized that: (i) HDC and key inflammatory mediators are more highly expressed in endometriotic lesions compared to control tissues; (ii) histamine receptors are predominantly found in immune cells and nerve fibers within the lesions, indicating increased neuroimmune activity; and (iii) individuals with endometriosis have higher systemic histamine levels compared to controls.

## 2. Results

### 2.1. Altered Expression Patterns of Histamine Receptors, HDC, and Inflammatory Genes in Endometriosis and Control Tissues

**Figure 1:**
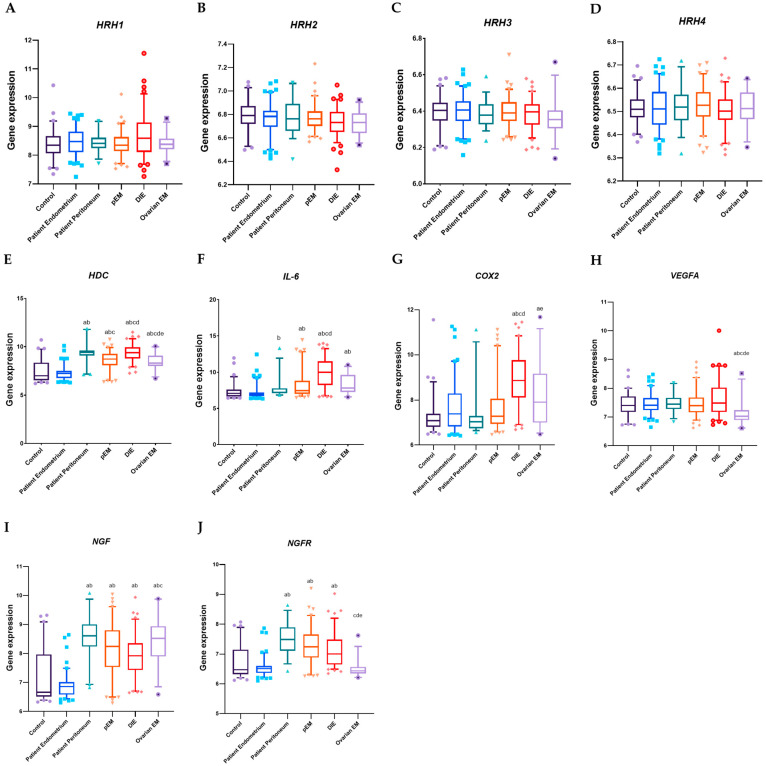
Altered Expression Patterns of Histamine Receptors, *HDC*, and Inflammatory Genes in Endometriosis and Control Tissues. Gene expression levels of *HRH1* (**A**), *HRH2* (**B**), *HRH3* (**C**), *HRH4* (**D**), *HDC* (**E**), *IL-6* (**F**), *COX2* (**G**), *VEGFA* (**H**), *NGF* (**I**), and *NGFR* (**J**) across the following groups: Control (N = 38), Patient Endometrium (N = 98), Patient Peritoneum (N = 37), peritoneal endometriosis (pEM; N = 78), deep infiltrating endometriosis (DIE; N = 88), and ovarian endometriosis (Ovarian EM; N = 29). Statistical analysis was performed using the Kruskal–Wallis test. a vs. Control group; b vs. Patient Endometrium; c vs. Patient Peritoneum; d vs. pEM; e vs. DIE.

Using gene expression data from the database provided by the University of Turku, we analyzed the expression levels of histamine receptors HRH1, HRH2, HRH3, and HRH4 across various endometriosis subtypes and control tissues (Figure 1A–D). No statistically significant differences were observed in the expression of these four histamine receptors among the groups. Descriptive statistics are presented as medians, along with 25th–75th percentiles, unless stated otherwise.

In contrast, several other genes showed significant differential expression. *HDC* expression (Figure 1E) was markedly elevated in patient peritoneum (9.40, 9.07–9.62; *p* < 0.0001), peritoneal endometriosis (8.71, 8.08–9.27; *p* < 0.0001), deep infiltrating endometriosis (9.38, 8.79–9.92; *p* < 0.0001), and ovarian endometriosis (8.33, 8.00–9.07; *p* = 0.0024) compared to control tissue (7.01, 6.54–8.37) and patient endometrium (7.24, 6.77–7.51; *p* < 0.0001 for both). Moreover, *HDC* levels in the patient’s peritoneum and deep infiltrating endometriosis were significantly higher than in peritoneal endometriosis (*p* = 0.0074 and *p* = 0.0002, respectively), and deep infiltrating endometriosis samples exhibited greater HDC expression than ovarian endometriosis (*p* = 0.0149).

*IL-6* expression (Figure 1F) was significantly increased in peritoneal endometriosis (7.44, 7.00–8.84; *p* = 0.0049), deep infiltrating endometriosis (10.00, 8.20–11.52; *p* < 0.0001), and ovarian endometriosis (7.80, 7.24–9.63; *p* = 0.0111) relative to control tissue (7.08, 6.66–7.60) and patient endometrium (6.90, 6.70–7.21). The patient’s peritoneum also displayed higher *IL-6* expression than the patient’s endometrium (7.22, 7.11–7.78 vs. 6.90, 6.70–7.21; *p* = 0.0070). Notably, deep infiltrating endometriosis samples showed the highest *IL-6* levels, significantly exceeding those of the patient’s peritoneum (*p* = 0.0001) and peritoneal endometriosis (*p* < 0.0001).

*COX2* expression peaked in deep infiltrating endometriosis samples (8.86, 8.12–9.76; Figure 1G), significantly surpassing control (7.08, 6.81–7.38; *p* < 0.0001), patient endometrium (7.38, 6.82–8.29; *p* < 0.0001), patient peritoneum (7.03, 6.75–7.29; *p* < 0.0001), peritoneal endometriosis (7.27, 6.94–8.06; *p* < 0.0001), and ovarian endometriosis (7.90, 6.99–9.17; *p* = 0.0356). Ovarian endometriosis also exhibited significantly higher *COX2* levels compared to controls (*p* = 0.0355).

Conversely, *VEGFA* expression (Figure 1H) was significantly lower in ovarian endometriosis (7.03, 6.89–7.24) relative to all other tissues, including control (7.40, 7.17–7.71; *p* = 0.0052), patient endometrium (7.40, 7.24–7.66; *p* = 0.0007), patient peritoneum (7.44, 7.28–7.66; *p* = 0.0023), peritoneal endometriosis (7.40, 7.16–7.68; *p* = 0.0040), and deep infiltrating endometriosis (7.48, 7.17–8.02; *p* < 0.0001).

*NGF* expression (Figure 1I) was significantly reduced in control (6.66, 6.51–7.96) and patient endometrium (6.86, 6.58–7.01) compared to all endometriosis subtypes (*p* < 0.0001). Within lesion types, deep infiltrating endometriosis exhibited lower *NGF* levels than the patient’s peritoneum (*p* = 0.0060).

*NGFR* expression (Figure 1J) was significantly upregulated in patient peritoneum (7.48, 7.10–7.89), peritoneal endometriosis (7.24, 6.88–7.66), and deep infiltrating endometriosis (6.99, 6.65–7.48) compared to control (6.47, 6.32–7.15) and patient endometrium (6.50, 6.36–6.59) (all *p* < 0.0001). Notably, ovarian endometriosis showed reduced *NGFR* expression (6.43, 6.34–6.56) relative to the other lesion types (*p* < 0.0001).

Spearman correlation analysis (Supplementary Material S1) revealed strong positive correlations between *HDC* and genes involved in inflammation, including *IL-6*, *COX2*, *NGF*, *NGFR*, and *VEGFA*, as well as with disease stage (all *p* < 0.001). *HDC* showed only a weak correlation with *HRH2* (*p* = 0.026). *HRH1* expression correlated significantly with *IL-6* and *COX2* (*p* < 0.001), and showed moderate associations with *VEGFA* (*p* = 0.002) and disease stage (*p* = 0.006). Minor correlations were found between *HRH2* and *VEGFA* (*p* = 0.012), and *HRH3* and disease stage (*p* = 0.027).

**Figure 2:**
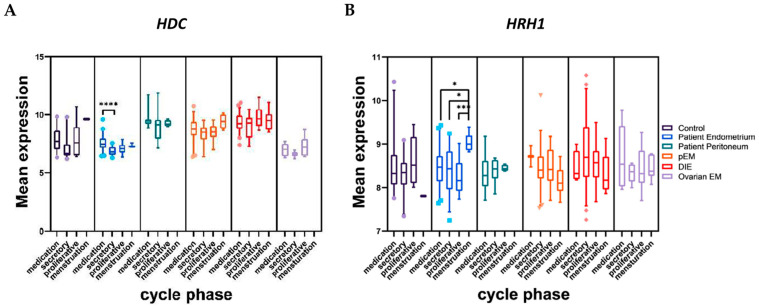
Influence of cycle phase and hormonal intake on mean gene expression of *HDC* (**A**) and *HRH1* (**B**). Gene expression was compared across the following tissue groups: Control (N = 38), Patient Endometrium (N = 98), Patient Peritoneum (N = 37), peritoneal endometriosis (pEM; N = 78), deep infiltrating endometriosis (DIE; N = 88), and ovarian endometriosis (Ovarian EM; N = 29). Within each group, patients were categorized according to menstrual cycle phase (menstruation, proliferative, secretory) and hormonal medication at the time of sample collection. Statistical analysis was performed using the Kruskal–Wallis test. *p* < 0.05 (*), *p* < 0.001 (***), *p* < 0.0001 (****).

We further investigated the influence of menstrual cycle phase (menstruation, secretory, proliferative) and hormonal medication on *HRH1-HRH4* and *HDC* expression within each group. To avoid any influence due to differing disease stages, the comparison was not made between all groups. Significant differences were observed only in the patient endometrium, where *HDC* expression was elevated in medicated patients compared to those in the secretory phase (7.46, 7.14–7.96 vs. 6.80, 6.53–7.21; *p* < 0.0001; Figure 2A). Similarly, *HRH1* expression in patient endometrium was highest during menstruation (8.99, 8.86–9.19) compared to secretory (8.43, 7.96–8.86; *p* = 0.0134), proliferative (8.16, 7.92–8.56; *p* = 0.0009), and medicated states (8.47, 8.14–8.73; *p* = 0.0185; Figure 2B). No significant variations were observed in *HRH2*, *HRH3*, or *HRH4* expression.

### 2.2. Histamine Receptor Distribution and Immune–Neural Interactions in Endometriosis

In the initial approach, double immunofluorescent staining with the established Protein Gene Product 9.5 (PGP9.5) and anti-histamine antibodies successfully validated the method. Histamine distribution was diffuse and laminar. However, all tissue slides exhibited autofluorescence. Subsequent attempts yielded improved results, with clearer differentiation. Based on these observations, we decided to investigate histamine receptors.

**Figure 3:**
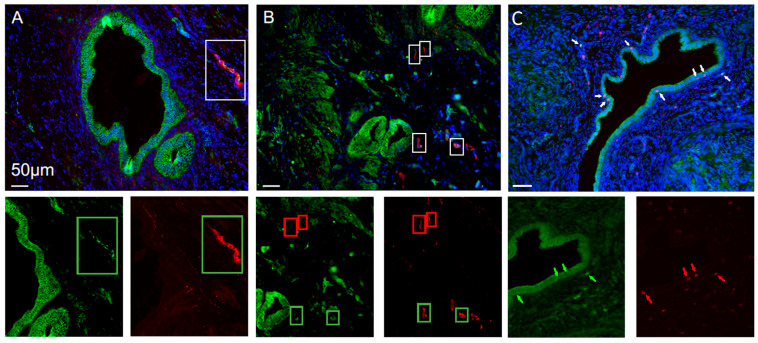
Representative immunofluorescence images demonstrating the colocalization of histamine receptors with nerve fibers or CD45-positive immune cells within endometriotic tissue. (**A**) Double immunostaining for HRH3 (green) and the neuronal marker PGP9.5 (red) showing HRH3-positive nerve fibers adjacent to a peritoneal endometriosis lesion. (**B**) HRH3-positive and -negative nerves located proximally and distally to the peritoneal lesion. (**C**) Double immunostaining for HRH1 (green) and the immune cell marker CD45 (red, marked by the arrows). Squares are marking the nerve fibers. Green squares and arrows indicate positive colocalization, while red squares and arrows indicate negative colocalization. Nuclei are counterstained with DAPI (blue). Images were acquired using a 20× objective. Scale bar: 50 µm for all panels.

**Figure 4:**
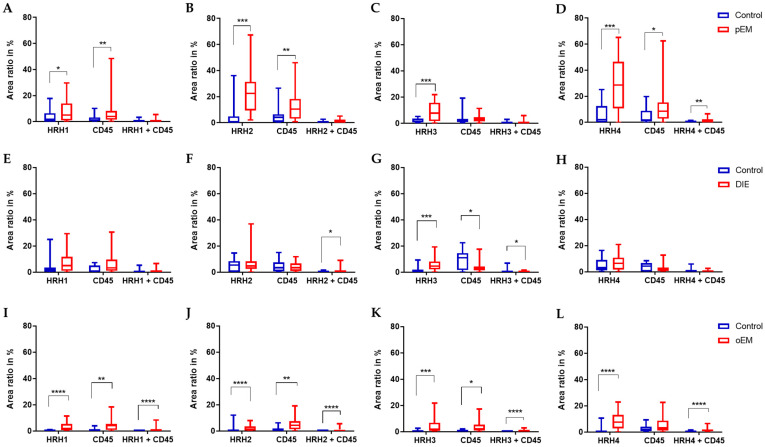
Expression and colocalization of histamine receptors (HRH1–HRH4) with the immune cell marker CD45 in different endometriosis subtypes. Box plots represent the area ratio (%) of HRH1–HRH4, CD45, and their colocalization (HRH + CD45) in control tissues (blue) compared with (**A**–**D**) peritoneal endometriosis (pEM), (**E**–**H**) deep infiltrating endometriosis (DIE), and (**I**–**L**) ovarian endometriosis (oEM). Quantification was performed based on immunofluorescence double staining for each receptor and CD45. The area ratio (%) reflects the proportion of marker-positive area in relation to the overall DAPI-positive area. Statistical analysis was carried out using the Mann–Whitney U test. *p* < 0.05 (*), *p* < 0.01 (**), *p* < 0.001 (***), *p* < 0.0001 (****).

All four histamine receptors (HRH1–HRH4) were detected in peritoneal endometriosis lesions (Figure 3). Quantitative analysis demonstrated a significantly higher expression of all four receptors in peritoneal endometriosis compared with control tissues (HRH1: 5.17, 1.04–13.96 vs. control: 1.80, 0.05–6.48; *p* = 0.01; HRH2: 22.48, 9.43–31.35 vs. control: 0.61, 0.18–4.89; *p* < 0.0001; HRH3: 7.76, 1.73–15.78 vs. control: 2.85, 0.79–3.69; *p* = 0.0045; HRH4: 28.67, 10.75–46.40 vs. control: 2.16, 0.15–12.51; *p* < 0.0001; Figure 4A–D). Expression of the receptors was localized in epithelial cells, immune cells, and endometriosis-associated nerve fibers.

CD45, a pan-leukocyte marker used to identify immune cells (including mast cells), showed increased expression in peritoneal endometriosis samples compared to controls (Figure 4A,B,D). It is important to note that CD45 expression in the control tissues showed some variability. This variation likely reflects the natural heterogeneity in immune-cell density across different pelvic tissue locations, even in non-diseased samples. Additionally, the number of controls available for each subtype was limited (n = 5), which may further accentuate apparent variability. Despite this, differences between endometriosis lesions and their respective controls remained consistent and statistically significant across analyses. Notably, colocalization of HRH4 with CD45-positive immune cells was significantly higher in peritoneal endometriosis tissues (0.60, 0.11–2.33 vs. 0.00, 0.00–0.63; *p* < 0.0001; Figure 4D), suggesting immune-associated histamine signaling within the lesions.

In deep infiltrating endometriosis, all four receptors were present, with HRH3 exhibiting markedly elevated expression compared to controls (4.67, 2.17–8.14 vs. 0.54, 0.31–1.92; *p* = 0.0001; Figure 4G). Interestingly, deep infiltrating endometriosis lesions displayed fewer CD45-positive cells (2.67, 1.38–4.46) than controls (10.94, 1.66–14.59, *p* = 0.045; Figure 4G). Nevertheless, colocalization of HRH2 and HRH3 with CD45 was higher in this endometriosis tissue (HRH2: 0.34, 0.00–1.55; HRH3: 0.23, 0.05–0.47) than in control tissues (both 0.00, 0.00–0.00; *p* = 0.034 and *p* = 0.012, respectively; Figure 4F,G). This pattern—reduced overall CD45-positive cell density combined with increased HRH2/HRH3 colocalization—suggests that deep infiltrating endometriosis may harbor a qualitatively distinct immune infiltrate compared with the other lesion types. Rather than a higher number of immune cells, DIE appears to contain immune cell subpopulations that more prominently express or interact with histamine receptors, indicating potential subtype-specific mechanisms of immune modulation.

Ovarian endometriosis lesions also expressed all histamine receptors, with significantly increased levels of HRH1 (1.85, 0.70–5.45 vs. 0.11, 0.04–0.36; *p* < 0.0001), HRH2 (1.40, 0.39–3.57 vs. 0.14, 0.01–0.19; *p* < 0.0001), HRH3 (1.97, 0.79–6.91 vs. 0.54, 0.12–1.30; *p* = 0.0008), and HRH4 (7.59, 2.91–13.11 vs. 0.11, 0.03–0.96; *p* < 0.0001) compared to their respective controls (Figure 4I–L). Immunostaining also revealed abundant CD45-positive cells and robust colocalization between all histamine receptors and CD45 in ovarian endometriosis (HRH1: 0.58, 0.29–1.05; HRH2: 0.39, 0.06–1.23; HRH3: 0.62, 0.18–1.16; HRH4: 1.24, 0.60–1.92; all *p* < 0.0001; Figure 4I–L), compared with minimal or absent colocalization in controls.

Because HRH3 is generally not expressed on immune cells, colocalization studies with the neuronal marker PGP9.5 were performed to assess histamine receptor presence in nerve fibers. Control tissues were consistently negative for HRH1–4 and PGP9.5 colocalization. In contrast, peritoneal endometriosis lesions exhibited HRH2-, HRH3-, and HRH4-positive nerve fibers in 41.97%, 49.37%, and 35.93% of all counted nerves, respectively. In deep infiltrating endometriosis tissues, HRH1-, HRH2-, and HRH3-positive nerve fibers were identified in 15.63%, 52.17%, and 28.95% of nerves, respectively, whereas ovarian endometriosis showed no receptor-positive nerve fibers.

To further characterize the neuronal subtypes involved, double immunostaining was performed using HRH1–4 and markers for sensory (substance P), sympathetic (tyrosine hydroxylase, TH), and parasympathetic (vasoactive intestinal peptide, VIP) fibers (Supplementary Material S2).

In peritoneal endometriosis, 17.64% of HRH2-positive nerves also expressed substance P, 44.12% expressed tyrosine hydroxylase, and 38.24% expressed VIP. HRH3-positive nerve bundles were predominantly associated with sensory fibers (71.79%), followed by parasympathetic (64.10%) and sympathetic (46.15%) fibers. HRH4-positive nerves also demonstrated broad distribution across neuronal subtypes, colocalizing with 34.78% of substance P-, 47.83% of tyrosine hydroxylase-, and 56.52% of VIP-positive fibers. These findings indicate that histamine receptors, particularly HRH2–HRH4, are expressed across multiple neural pathways in peritoneal endometriosis, suggesting potential histamine-mediated modulation of both sensory and autonomic innervation.

In deep infiltrating endometriosis, 20% of HRH1-positive nerves colocalized with substance P, and 40% showed colocalization with both tyrosine hydroxylase and VIP. HRH2-positive nerves displayed a similar distribution pattern, with an average of 33.33% colocalization across the three neuronal markers. More specifically, HRH2-positive fibers overlapped with 36.36% of sensory and sympathetic markers and 45.45% of parasympathetic markers. This pattern suggests that histamine signaling via HRH1 and HRH2 may influence both nociceptive and autonomic components within deep infiltrating endometriosis lesions.

### 2.3. Comparative Analysis of Histamine Concentrations in Peritoneal Fluid and Serum from Endometriosis Patients and Controls

**Figure 5:**
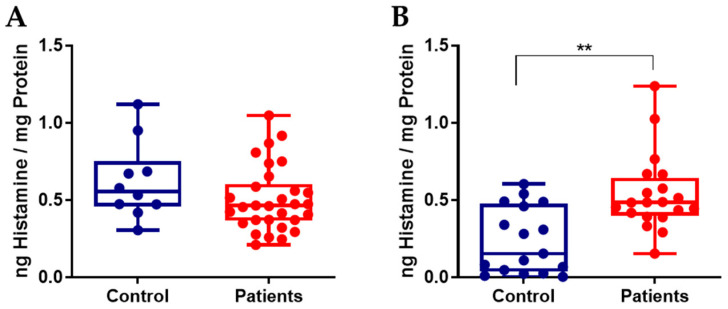
Histamine levels in peritoneal fluid (**A**) and blood serum (**B**) from control individuals and endometriosis patients. Values are normalized to protein levels (ng/mg protein). Sample sizes were n = 10 for controls and n = 30 for endometriosis patients in peritoneal fluid, and n = 10 for controls and n = 30 for endometriosis patients in serum. Statistical analysis was performed using the non-parametric Mann–Whitney U test (two-tailed); *p* < 0.01 (**).

Histamine concentrations were quantified using a competitive ELISA. In peritoneal fluid samples (Figure 5A), median histamine levels normalized to total protein measured from the same sample were 0.554 ng/mg (0.458–0.750) in the control group (n = 10) and 0.463 ng/mg (0.365–0.602) in patients with endometriosis (n = 30), with values comparable between groups (*p* = 0.1314).

In contrast, serum samples (Figure 5B) from patients with endometriosis exhibited markedly higher histamine concentrations normalized to total protein (0.484 ng/mg, 0.398–0.643) compared with controls (0.153 ng/mg, 0.036–0.474; *p* = 0.0014; median difference = 0.3305), indicating a significant systemic increase in circulating histamine levels associated with endometriosis.

### 2.4. Methylhistamine Measurement in the Urine of Patients and Controls

**Figure 6:**
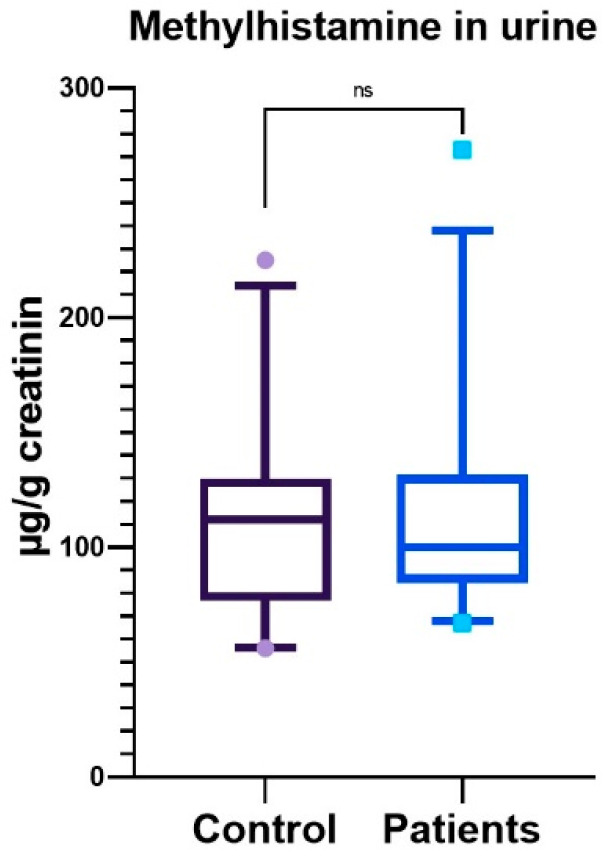
Methylhistamine levels in urine samples from control individuals and endometriosis patients. Values are normalized to creatinine and expressed in µg/g creatinine. Statistical analysis was performed using the non-parametric Mann–Whitney U test (two-tailed); no significant differences were observed.

The concentration of methylhistamine in the spontaneous urine of women with endometriosis was compared to that of women without the disease. No significant difference between the two groups was found (109.6 ± 38.94 μg/g creatinine vs. 113.0 ± 45.55 μg/g creatinine; *p* = 0.9534). The levels of methylhistamine were measured in comparison to the levels of creatinine, which is a waste product produced by the body at a relatively constant rate and excreted in the urine. This measurement helps to normalize the results, accounting for variations in urine concentration. This is important because it enables a more accurate assessment of methylhistamine levels, which can be influenced by factors such as hydration status and kidney function [30,31]. The reference range for methylhistamine concentration to creatinine, as provided by Labor Berlin, is between 34.0 and 177.0 μg/g creatinine. Above this range was one control patient with 225 μg/g creatinine and two endometriosis patients with 273 and 223 μg/g creatinine, respectively. Figure 6 shows the box plot of controls and patients.

To further investigate the potential clinical relevance, we assessed correlations between histamine activity, quantified via urinary methylhistamine excretion, and patient age, acknowledging the established inverse relationship between age and endometriosis risk. Additionally, we examined associations with hallmark symptoms of endometriosis, including cyclic pelvic pain, dyschezia, and abdominal distension (commonly referred to as endobelly). Spearman’s rank correlation analysis (Supplementary Material S3) did not identify any statistically significant associations between methylhistamine levels and these clinical variables, suggesting that histamine turnover, as reflected by urinary methylhistamine, may not be directly linked to age or symptom severity in this cohort. Taken together, these findings suggest that urinary methylhistamine is unlikely to be a useful biomarker for endometriosis in this cohort.

## 3. Discussion

This study provides the first comprehensive characterization of histamine receptor expression and localization across the three main subtypes of endometriosis—peritoneal, deep infiltrating, and ovarian—and delineates their association with immune and neuronal markers. By integrating gene expression analyses with receptor immunolocalization and co-staining for immune and neuronal subtypes, we demonstrate that histamine signaling is a pervasive feature of the endometriotic microenvironment, extending beyond mast cell–mediated inflammation to encompass neuroimmune crosstalk and potentially nociceptive modulation.

The detection of histamine in tissue samples using immunohistochemistry and immunofluorescence posed significant challenges. Both staining techniques yielded non-descriptive results, with the polyclonal histamine antibody showing a widespread, uniform distribution across the endometriosis tissue, suggesting a ubiquitous presence of histamine. Despite optimizing standard protocols, successful histamine staining in tissue has not yet been achieved, as confirmed by dermatologists and pathologists at Charité. Previous attempts to stain mast cells with histamine in the 1960s also proved unsuccessful, underscoring the difficulty of this method [32,33].

Alternative methods were investigated after direct histamine staining failed in endometriosis tissue. Indirect methods such as ELISA, HDC expression analysis, and mast cell staining (e.g., CD45-positive cells) provide valuable information, but direct tissue analysis remains essential to expand our understanding of histamine’s role in the pathophysiology of endometriosis.

Our data show significantly elevated expression of HDC, the enzyme responsible for histamine synthesis, in all endometriosis lesion types compared to controls. This upregulation was accompanied by increased expression of inflammatory mediators, including IL-6 and COX2, as well as NGF and NGFR, which are known to mediate neurogenic inflammation and pain sensitization [7,34]. Strong correlations between HDC and these proinflammatory and neurotrophic factors are observed; however, it is important to note that correlation does not prove causation. Other upstream factors, such as estrogen or local hypoxia, could contribute to the observed patterns. Nevertheless, these findings are consistent with a model in which histamine may contribute to the chronic inflammatory milieu of endometriosis by amplifying cytokine release and promoting neuroangiogenesis [35,36]. They also align with previous reports of increased mast cell density and degranulation within endometriotic lesions and their spatial association with sensory nerve fibers [9,11,22], suggesting that histamine release serves as a key interface between immune activation and nociceptive signaling.

Although transcriptomic analysis did not reveal significant differences in histamine receptor mRNA expression between tissue types, immunofluorescence staining revealed robust receptor protein expression across all lesion subtypes, suggesting potential post-transcriptional regulation or localized receptor stabilization. All four receptors (HRH1-4) were detected with the strongest signals in peritoneal and ovarian endometriosis, in epithelial cells, endometriosis-associated immune cells, and nerve fibers associated with endometriosis. This discrepancy between mRNA and protein levels is consistent with previous findings showing that histamine receptor abundance and signaling can be modulated by post-transcriptional mechanisms (e.g., microRNA regulation, mRNA stability) and post-translational processes such as receptor trafficking, membrane recycling, and ligand-dependent stabilization in inflammatory tissues [37,38,39]. These studies support the plausibility of post-translational control contributing to receptor expression patterns observed in endometriosis lesions.

Epithelial cells can express histamine receptors and trigger pro-inflammatory cytokine release, with HRH1 being the most extensively studied. In bronchial and nasal epithelial cells, HRH1 activation induces cytokines such as IL-8 and GM-CSF and modulates tight junction proteins, implicating HRH1 in epithelial-driven inflammatory responses [40,41]. HRH2 appears to play a weaker or indirect role in epithelial cytokine induction, primarily by modulating immune cells such as dendritic cells [42]. HRH3 is primarily neuronal, with limited evidence for epithelial inflammation, though some studies suggest it may contribute via crosstalk with HRH1 and HRH2 [43]. H4HR modulates immune responses, but direct evidence in epithelial cells is scarce; it is known to regulate cytokine secretion in monocytes and brain endothelial cells [44,45]. Notably, HRH4 colocalized with CD45-positive immune cells in peritoneal endometriosis, suggesting a role in leukocyte recruitment and activation. In contrast, deep infiltrating lesions showed stronger colocalization of HRH2 and HRH3 with immune markers, indicating receptor–subtype–specific functions within distinct lesion microenvironments.

A major novel finding of this study is the demonstration of histamine receptor expression in peripheral nerve fibers within endometriotic lesions. Specifically, HRH2 and HRH4 were detected in nerve fibers of peritoneal endometriosis, whereas HRH1–HRH3 were expressed in nerve fibers of deep infiltrating endometriosis. Control tissues lacked histamine receptor colocalization with the neuronal marker PGP9.5, highlighting the disease-specific nature of this phenomenon. This is supported by a recent study [13], which showed that endometriotic cell–derived human β-defensin-2 (HBD-2) stimulates mast cell histamine release via MRGPRX2 (Mas-Related G-Protein Coupled Receptor X2), which then sensitizes TRPV1 through HRH1 in pain-sensory neurons. Correspondingly, mice deficient in Mrgprb2, exhibit reduced hyperalgesia and smaller endometriotic lesions.

Double labeling with neuronal subtype markers revealed that histamine receptor–positive fibers were distributed across sensory (substance P), sympathetic (TH), and parasympathetic (VIP) fibers, with HRH3 and HRH4 showing the broadest colocalization profiles. In peritoneal endometriosis, the majority of HRH3-positive nerves were sensory, supporting the role of histamine in nociceptive sensitization. The presence of HRH2- and HRH3-positive fibers within both sensory and autonomic systems in deep infiltrating endometriosis suggests that histamine may also contribute to visceral dysregulation and autonomic dysfunction, components increasingly recognized in endometriosis-associated pain syndromes [1,7].

Histamine quantification revealed distinct local and systemic patterns in endometriosis. Peritoneal fluid levels were comparable between patients and controls, suggesting tightly regulated local histamine activity. In contrast, serum histamine was significantly elevated, indicating systemic dysregulation that could contribute to inflammation, neuronal sensitization, and endometriosis-associated pain [46,47]. A similar observation was reported by Oraziv and collaborators in 2017 [48]. The discrepancy between elevated serum and unchanged peritoneal histamine levels likely reflects differences in histamine metabolism and compartmental regulation. Within the peritoneal cavity, histamine is rapidly degraded by enzymes such as histamine-N-methyltransferase (HNMT) and diamine oxidase (DAO), leading to low measurable concentrations despite strong local HDC expression and mast-cell activation [49,50]. Conversely, chronic activation of mast cells, macrophages, and epithelial cells across multiple endometriotic foci may drive cumulative systemic histamine release, reflected by the elevated circulating levels [14,15]. Locally, histamine may also bind to high-affinity receptors (HRH3 and HRH4) on immune and nerve cells, further reducing the pool of free histamine detectable in peritoneal fluid. It is important to note that serum histamine levels are influenced by various systemic factors, including diet and allergies, even though we applied exclusion criteria and dietary instructions to minimize these influences. Together with upregulation of inflammatory mediators such as IL-6, COX-2, and NGF, these findings support a model of widespread immune activation and systemic mast-cell priming, indicating that histamine signaling in endometriosis is both locally active and systemically amplified. Urinary methylhistamine, normalized to creatinine, did not differ between groups and showed no correlation with age or key symptoms, suggesting that it may not reliably reflect disease activity or histamine turnover in endometriosis [30]. Overall, these findings support a model in which systemic histamine elevation contributes to the chronic inflammatory and neurogenic milieu in endometriosis. Future studies should investigate the cellular sources of circulating histamine, receptor-specific signaling, and correlations with clinical outcomes, mast cell infiltration, and neuronal sensitization to better elucidate histamine’s mechanistic role in endometriosis-associated pain.

While our study provides robust histological and molecular evidence of histamine receptor involvement in endometriosis, some limitations should be acknowledged. The sample size per lesion subtype was relatively small, and receptor quantification was semi-quantitative. Moreover, we did not perform functional assays to assess downstream signaling or receptor-specific pharmacologic responses, which would be required to confirm receptor activity in endometriotic lesions. The cross-sectional design also limits our ability to infer associations between histamine-related changes and long-term pain outcomes. Furthermore, the public GEO dataset used for transcriptomic validation may be affected by batch effects or sample heterogeneity, which could influence gene expression comparisons. Additionally, exploring interactions between histamine receptors and other pain-related mediators, such as TRPV1 and CGRP, could clarify the mechanisms by which histamine contributes to sensory hypersensitivity [13]. Future studies should evaluate the therapeutic potential of histamine receptor antagonists and mast-cell stabilizers in preclinical models of endometriosis to determine whether targeting this pathway alleviates inflammation or pain. Moreover, integrating single-cell or spatial transcriptomic approaches would enable precise identification of histamine-responsive cell populations within lesions and reveal cell-type–specific signaling programs that cannot be resolved with bulk analyses.

## 4. Materials and Methods

### 4.1. Patients and Samples

This study comprised four separate analyses, each involving different cohorts of women with and without endometriosis. All participants provided written informed consent, and the study was conducted in accordance with the Declaration of Helsinki and received approval from the Institutional Review Board of the Charité University Medical Centre (Ethic vote EA4/053/25).

For the immunofluorescence staining, tissue samples were collected during laparoscopic surgery at Charité—Universitätsmedizin Berlin. Patients were selected based on clinical, intraoperative, and subsequent histopathologic findings. Control samples were collected from women without endometriosis, who had undergone laparoscopy for benign gynecological presentations such as non-endometriosis associated with ovarian cysts, uterine fibroids, Hydrosalpingx, pelvic pain, or the unfulfilled wish to have children. A total of 10 samples were analyzed for each of the three endometriosis subtypes: peritoneal endometriosis, deep infiltrating endometriosis, and ovarian endometriosis. Additionally, five location-matched control samples were included for each subtype, resulting in a total of 30 endometriosis samples and 15 control samples; control tissues were peritoneum for peritoneal endometriosis, bladder and colon for DIE, and ovary for ovarian endometriosis.

The gene expression analysis utilized publicly available data, including a total of 330 patients with endometriosis and 62 control participants. Further details concerning sample types, methodology, and clinical metadata are described in the relevant analysis sections.

For the analysis of histamine concentrations in serum and peritoneal fluid, samples were collected from women with and without endometriosis. Serum samples were obtained either from patients with endometriosis (n = 20) before anesthesia induction on the day of laparoscopic surgery at Charité—Universitätsmedizin Berlin or from outpatients diagnosed with endometriosis (n = 20). Control serum samples (n = 20) were provided by the institutional blood donation bank or obtained from healthy volunteers recruited via an announcement on the Charité intranet and personal networks. Peritoneal fluid samples were collected intraoperatively during laparoscopy from patients with endometriosis (n = 30) and from women without endometriosis (n = 10) undergoing surgery for benign gynecological conditions such as ovarian cysts, uterine fibroids, hydrosalpinx, pelvic pain, or infertility. All samples were immediately processed, aliquoted, and stored at −80 °C until analysis.

For the urinary methylhistamine quantification, a total of 60 participants were recruited: 35 patients diagnosed with endometriosis from the Endometriosis outpatient clinic at Charité—Universitätsmedizin Berlin, and 25 control participants recruited via an announcement on the Charité intranet and personal networks. Women with a confirmed diagnosis of endometriosis were included in the patient group. Exclusion criteria for all participants included pregnancy, postmenopausal status, malignant disease, atopic dermatitis, neurodermatitis, and known allergies.

All participants were instructed to avoid histamine-rich foods (e.g., aged cheese, smoked meats, seafood, fermented products, wine, beer, coffee, and black tea) for 24 h before urine collection. A 24 h dietary questionnaire, along with general questions regarding menstrual cycle, hormonal intake, medical history, medication, and supplement use, was completed by all participants. Additionally, patients with endometriosis filled out a brief pain questionnaire. All questionnaires were administered in German.

The characteristics of the patients for all the different analyses are listed in Supplementary Material S4.

### 4.2. Analysis of Endometriosis-Associated Gene Expression and Clinical Features Using GEO Dataset GSE141549

Through collaboration with the University of Turku, we evaluated gene expression and phenotypic data from patients with endometriosis and those without using the Gene Expression Omnibus (GEO) database GSE141549 [51,52]. All samples underwent hybridization utilizing the Illumina Human 6 V2 microarray platform. In the present study, we re-analyzed the processed expression data available in GEO, which had been background-corrected, quantile-normalized, and log2-transformed as described previously by Gabriel et al. [51].

A total of 330 patients with endometriosis and 62 controls were included. Tissues analyzed from patients comprised endometrium, peritoneum, peritoneal endometriosis, deep infiltrating endometriosis, and ovarian endometriosis. For women without endometriosis, only endometrial and peritoneal samples were collected and used as control tissues. Additional clinical information, including age, ASRM disease stage, hormonal treatment, and menstrual cycle phase, was retrieved from the GEO database. Hormonal treatments administered within three months prior to surgery were recorded during clinical examination and were used to classify samples as treated or untreated.

### 4.3. Immunofluorescence Staining

All biopsies were immediately fixed in buffered formalin (4%) for at least 12 h and then embedded in paraffin. Sections with a thickness of 2 μm were cut for immunofluorescence staining, utilizing antibodies against histamine, HRH1-4 receptors, CD45, PGP 9.5, substance P, tyrosine hydroxylase (TH), and VIP (see Table 1).

Slides were first deparaffinized and rehydrated by submersing in xylene (10 min, twice), followed by 100% ethanol (3 min, twice), 96% ethanol (3 min), 80% ethanol (3 min), and 70% ethanol (3 min), and finally washed in dH2O for 5 min. Antigen retrieval was then performed by incubating slides in Target 9 buffer at 98 °C for 20 min, followed by gradual cooling in the same buffer for 30 min at room temperature. For intracellular antigens, slides were permeabilized with 0.5% Triton X-100 in 1× PBS for 20 min. Slides were washed with 0.1% Tween-20 in PBS for 5 min and then twice with PBS for 2 min.

Samples were blocked with 2% normal goat serum for 1 h at room temperature and incubated with the appropriate primary antibodies overnight at 4 °C. Slides were then washed with 0.1% Tween in 1× PBS for 5 min, followed by two washes with 1× PBS for 2 min each. Samples were incubated with the appropriate secondary antibodies for 1 h at room temperature and handled under minimal light from this point onward. The previous wash step was repeated. Nuclei were visualized by incubating with DAPI (1:20) for 15 min, followed by three washes in dH2O for 2 min each. Slides were mounted with VectaMount^®^ AQ Aqueous Mounting Medium. Negative control sections were prepared by omitting the specific primary antibody. Colon, stomach, brain, and skin tissue sections served as positive controls for the receptors. Staining was visualized using a BZ-X80 Fluorescence microscope (Keyence Germany, Frankfurt am Main, Germany). Photomicrographs were acquired at 20× magnification and processed with the BZ-X800 Viewer software.

The density was determined using the software’s hybrid cell count analyzer (Keyence Germany, Frankfurt am Main, Germany). The extraction area (%) of each corresponding marker was measured using the automatically set threshold relative to the target area of DAPI-positive cells. To account for sample-specific autofluorescence, the automatically determined threshold was manually adjusted when necessary for individual samples to optimally distinguish the true signal from the background. For nerve density analysis (PGP 9.5), one complete slide per patient was evaluated, and all nerves present on the entire slide were counted. Staining density was sequentially assessed by two investigators blinded to sample identities. Each patient was assigned a code, which remained concealed until the analysis was completed at the end of the study. In cases of disagreement, both observers repeated the analysis together to reach a consensus.

### 4.4. Urinary Methylhistamine Quantification by HPLC in Endometriosis and Control Subjects

Methylhistamine, a degradation product of histamine excreted in urine, was measured in endometriosis patients and healthy controls. Spot (spontaneous) urine samples were collected from each participant. The collection was not standardized to a specific phase of the menstrual cycle. Urine was collected in a urine pot and put in the fridge if not processed within one hour. 1.5 mL of urine was transferred into Eppendorf tubes and frozen at −20 °C until it was sent to Labor Berlin. Methylhistamine concentrations were quantified by high-performance liquid chromatography (HPLC) with appropriate sample preparation and detection, following established analytical protocols for amine metabolite quantification in urine (e.g., separation of histamine and methylhistamine by cation-exchange or reversed-phase HPLC methods with fluorometric or mass spectrometric detection) [53].

### 4.5. Histamine Measurement in Peritoneal Fluid and Serum by ELISA

Histamine levels in peritoneal fluid and serum samples were quantified using a competitive enzyme-linked immunosorbent assay (ELISA) kit specific for histamine (Abcam, Cambridge, UK, catalog number ab213975), following the manufacturer’s protocol. Briefly, samples and histamine standards were added to wells coated with a histamine conjugate. A histamine-specific antibody was then introduced, which competes with free histamine in the sample and the immobilized histamine conjugate on the plate.

After incubation, wells were washed to remove unbound components. A secondary antibody conjugated to horseradish peroxidase (HRP) was then added, followed by the substrate solution. The enzymatic reaction produced a color change inversely proportional to the histamine concentration in the samples. Absorbance was measured at 450 nm using a microplate reader (SpectraMaz i3x, Molecular Devices, Munich, Germany).

Histamine concentrations were determined by interpolation from a standard curve generated with known histamine concentrations. All samples and standards were assayed in duplicate to ensure accuracy and reproducibility.

### 4.6. Statistics

Statistical analyses were performed using GraphPad Prism version 8.4.2 and IBM SPSS for Windows version 29.0.0.0. Data distributions were evaluated with appropriate normality tests to guide the selection of parametric or nonparametric methods. Descriptive statistics are presented as medians, along with 25th–75th percentiles, unless stated otherwise. Group comparisons between two independent samples were conducted using the Mann–Whitney U test. For comparisons involving more than two groups, the Kruskal–Wallis test was followed by Dunn’s post hoc correction for multiple comparisons. Nonparametric correlations were assessed using Spearman’s rank correlation coefficient. Categorical variables were analyzed with Chi-square or Fisher’s exact tests, as appropriate. Statistical significance thresholds were set at * *p* < 0.05, ** *p* < 0.01, *** *p* < 0.001, and **** *p* < 0.0001; non-significant results are denoted as ns.

## 5. Conclusions

This study identifies histamine-related sensitization in endometriosis with high HDC-expression and the corresponding histamine receptors HRH1–HRH4 as integral components of the endometriotic neuroimmune interface. Their widespread expression across immune cells and nerve fibers supports a model in which histamine drives both inflammatory and neural activation within lesions, thereby contributing to chronic pain and local immune dysregulation. These findings establish histamine signaling as a potential therapeutic target in endometriosis, bridging inflammatory and neuronal pathways and offering new avenues for mechanism-based treatment strategies.

## Figures and Tables

**Table 1:** List of the antibodies and dilutions used in this study.

Marker/Receptor Antibody	Dilution	Company
Anti-Histamine Rabbit Polyclonal	1:50 to 1:1000	Invitrogen, Waltham, MA, USA (PA5-119564)
Anti-Histamine H1 Receptor/HRH1 Rabbit Polyclonal	1:200	Alomone Labs, Jerusalem, Israel (AHR-006)
Anti-Histamine H2 Receptor/HRH2 Rabbit Polyclonal	1:200	Alomone Labs, Jerusalem, Israel (AHR-002)
Anti-Histamine H3 Receptor/HRH3 Rabbit Polyclonal	1:200	OriGene, Rockville, MD, USA (TA377221)
Anti-Histamine H4 Receptor/HRH4 Rabbit Polyclonal	1:200	OriGene, Rockville, MD, USA (SP4670P)
Anti-CD45 Mouse Monoclonal	1:500	Agilent Dako, Glostrup, Denmark (M0701)
Anti-UCH-L1/PGP9.5 (Protein gene product 9.5) Chicken Polyclonal	1:500	Novus, Los Angeles, CA, USA (NB110-58872)
Monoclonal mouse anti-TH (Tyrosine hydroxylase)	1:100	Sigma, St. Louis, MO, USA (T2928)
Monoclonal mouse anti-VIP (Vasoactive intestinal peptide)	1:100	Santa Cruz, Los Angeles, CA, USA (sc-25347)
Monoclonal rat anti-SP (Substance P)	1:500	Santa Cruz, Los Angeles, CA, USA (sc-21715)

## Data Availability

The original contributions presented in this study are included in the article and Supplementary Materials. Further inquiries can be directed to the corresponding author.

## Supplementary Materials

The following supporting information can be downloaded at: https://www.mdpi.com/article/10.3390/ijms27010212/s1.

## Abbreviations

The following abbreviations are used in this manuscript:

ASRM	American Society for Reproductive Medicine
BEAS-2B	Human bronchial epithelial cell line
CD45	Cluster of Differentiation 45
CGRP	Calcitonin Gene-Related Peptide
CNS	Central Nervous System
COX2	Cyclooxygenase-2
Cyt	Cytokine
DAPI	4′,6-Diamidino-2-Phenylindole
DAO	Diamine Oxidase
DIE	Deep Infiltrating Endometriosis
EC	Endothelial Cells
ELISA	Enzyme-Linked Immunosorbent Assay
EM	Endometriosis
GEO	Gene Expression Omnibus
GM-CSF	Granulocyte-Macrophage Colony-Stimulating Factor
HBD-2	Human β-Defensin-2
HDC	Histidine Decarboxylase
HNMT	Histamine-N-Methyltransferase
HPLC	High-Performance Liquid Chromatography
HRH1	Histamine Receptor H1
HRH2	Histamine Receptor H2
HRH3	Histamine Receptor H3
HRH4	Histamine Receptor H4
IL-6	Interleukin-6
IL-8	Interleukin-8
MRGPRX2	Mas-Related G-Protein Coupled Receptor X2
NGF	Nerve Growth Factor
NGFR	Nerve Growth Factor Receptor
NK cells	Natural Killer Cells
oEM	Ovarian Endometriosis
pEM	Peritoneal Endometriosis
PGP9.5/UCH-L1	Protein Gene Product 9.5
PKC	Protein Kinase C
PNS	Peripheral Nervous System
SP	Substance P
TH	Tyrosine Hydroxylase
TNF-α	Tumor Necrosis Factor-alpha
TRPV1	Transient Receptor Potential Vanilloid 1
VIP	Vasoactive Intestinal Peptide
VEGFA	Vascular Endothelial Growth Factor A
