# Supplementary material for: The Histamine-Associated Inflammatory Landscape of Endometriosis: Molecular Profiling of HDC, HRH1-HRH4, and Cytokines Across Lesion Subtypes
Source: Int J Mol Sci. 2025 Dec 24;27(1):212. doi: 10.3390/ijms27010212 (PMC12785993; doi:10.3390/ijms27010212)
Supplement: Supplementary file 1 [file ijms-27-00212-s001.zip › ijms-4029711-supplementary/Suppl. Material S1.pdf]

Supplementary Material S1

Table S1. Spearman's correlation analysis on *HDC, HRH1, HRH2, HRH3, HRH4, IL-6, COX2, NGF, NGFR, VEGFA*, age, disease stage, and cycle. The table depicts the genes *HDC, HRH1, HRH2, HRH3,* and *HRH4* correlated with all named parameters. Outputs are p-values and Rho. p < 0.05 (*), p < 0.01 (**), p < 0.001 (***), p < 0.0001 (****).

Gene	Correlated with	Rho	*P* value
HDC	*HRH1*	0.079	0.112
	HRH2	**- 0.110**	**0.026***
	HRH3	- 0.030	0.549
	HRH4	0.060	0.230
	IL-6	**0.602**	**< 0.001*****
	COX2	**0.297**	**< 0.001*****
	NGF	**0.726**	**< 0.001*****
	NGFR	**0.650**	**< 0.001*****
	VEGFA	**0.169**	**< 0.001*****
	Age	- 0.088	0.076
	Disease stage	**0.230**	**< 0.001*****
	Menstrual cycle	- 0.094	0.059
HRH1	*HRH2*	0.012	0.805
	HRH3	0.035	0.485
	HRH4	0.003	0.945
	IL-6	**0.283**	**< 0.001*****
	COX2	**0.176**	**< 0.001*****
	NGF	- 0.035	0.483
	NGFR	- 0.012	0.817
	VEGFA	**0.155**	**0.002****
	Age	- 0.039	0.429
	Disease stage	**0.136**	**0.006****
	Menstrual cycle	- 0.028	0.578
HRH2	*HRH3*	0.006	0.908
	HRH4	**0.106**	**0.033***
	IL-6	- 0.085	0.085
	COX2	- 0.056	0.261
	NGF	- 0.057	0.248
	NGFR	0.036	0.471
	VEGFA	**- 0.124**	**0.012***
	Age	- 0.015	0.755
	Disease stage	- 0.066	0.184
	Menstrual cycle	- 0.033	0.512
HRH3	*HRH4*	0.031	0.533
	IL-6	- 0.004	0.936
	COX2	- 0.007	0.894
	NGF	0.037	0.489
	NGFR	0.003	0.955
	VEGFA	0.076	0.125
	Age	- 0.090	0.070
	Disease stage	**- 0.110**	**0.027***
	Menstrual cycle	- 0.041	0.409
HRH4	*IL-6*	0.004	0.933
	COX2	0.003	0.957
	NGF	0.096	0.053

Gene	Correlated with	Rho	*P* value
	NGFR	0.077	0.123
	VEGFA	- 0.002	0.968
	Age	- 0.081	0.102
	Disease stage	- 0.037	0.455
	Menstrual cycle	- 0.019	0.709
