# Supplementary material for: The Histamine-Associated Inflammatory Landscape of Endometriosis: Molecular Profiling of HDC, HRH1-HRH4, and Cytokines Across Lesion Subtypes
Source: Int J Mol Sci. 2025 Dec 24;27(1):212. doi: 10.3390/ijms27010212 (PMC12785993; doi:10.3390/ijms27010212)
Supplement: Supplementary file 1 [file ijms-27-00212-s001.zip › ijms-4029711-supplementary/Suppl. Material S2.pdf]

Supplementary Material S2

Table S2. Counts of HRH-Positive and Total Nerve Fibers Across Endometriosis Subtypes and Controls

Sample type	Nerve Count	HRH1 positive		Nerve Count	HRH2 positive		Nerve Count	HRH3 positive		Nerve Count	HRH4 positive	
		number	%		number	%		number	%		number	%
pEM	72	0	0	81	34	72,59	79	39	50,07	64	23	61,50
Control	8	0	0	3	0	0	4	0	0	11	0	0
DIE	32	5	32,14	23	12	56,66	38	11	46,29	33	0	0
Control	8	0	0	15	0	0	22	0	0	10	0	0
oEM	18	0	0	11	0	0	12	0	0	10	0	0
Control	50	0	0	33	0	0	37	0	0	31	0	0

Sample type	HRH1	SP	TH	VIP	HRH2	SP	TH	VIP	HRH3	SP	TH	VIP	HRH4	SP	TH	VIP
		Positive (%)				Positive (%)				Positive (%)				Positive (%)		
pEM	0	0	0	0	72,59	21,82	50,58	37,253	50,07	79,99	62,08	80,42	61,50	31,55	60,72	64,28
DIE	32,14	12,50	62,50	62,50	56,66	62,50	62,50	62,50	46,29	26,67	35,55	46,67	0	0	0	0
oEM	0	0	0	0	0	0	0	0	0	0	0	0	0	0	0	0

pEM: peritoneal endometriosis; DIE: deep infiltrating endometriosis; oEM: ovarian endometriosis
