# Supplementary material for: The Histamine-Associated Inflammatory Landscape of Endometriosis: Molecular Profiling of HDC, HRH1-HRH4, and Cytokines Across Lesion Subtypes
Source: Int J Mol Sci. 2025 Dec 24;27(1):212. doi: 10.3390/ijms27010212 (PMC12785993; doi:10.3390/ijms27010212)
Supplement: Supplementary file 1 [file ijms-27-00212-s001.zip › ijms-4029711-supplementary/Suppl. Material S3.pdf]

Supplementary Material S3

Table S3. Correlation analysis

**Correlations**	**Rho**	***P*-value**
Methylhistamine measurements (μg/g creatinine)		
Age	-0.191	0.151
Cyclic pain	0.032	0.860
Cyclic pelvic pain	-0.128	0.493
Acyclic pelvic pain	-0.085	0.639
Dyspareunia	-0.102	0.578
Dyschezia	-0.030	0.870
Dysuria	0.050	0.783
Endobelly	0.018	0.923
