# Supplementary material for: The Histamine-Associated Inflammatory Landscape of Endometriosis: Molecular Profiling of HDC, HRH1-HRH4, and Cytokines Across Lesion Subtypes
Source: Int J Mol Sci. 2025 Dec 24;27(1):212. doi: 10.3390/ijms27010212 (PMC12785993; doi:10.3390/ijms27010212)
Supplement: Supplementary file 1 [file ijms-27-00212-s001.zip › ijms-4029711-supplementary/Suppl. Material S4.pdf]

Supplementary Material S4

Table S4. Patient and control clinical characteristics from individuals recruited for immunofluorescence staining.

	Patient group (n = 30)	Control group (n = 20)
Mean age ± SD (range)	30.3 ± 7.49 (18 – 47)	37.9 ± 5.62 (27 – 46)
Hormonal intake		
Yes	7	1
No	2	3
No data	21	15
Disease stage		
rASRM I-II	12	-
rASRM III-IV	18	-

Table S5. Patient and control clinical characteristics from individuals recruited for ELISA.

Serum samples	Patient group (n = 20)	Control group (n = 20)
Mean age ± SD (range)	30.3 ± 6.45 (20 – 40)	32.7 ± 3.05 (30 – 36)*
Hormonal intake		
Yes	1	-
No	-	-
No data	19	20
Disease stage		
rASRM I-II	4	-
rASRM III-IV	19	-

*Unfortunately, we were able to obtain age information for only 4 of the 20 control subjects.

Peritoneal fluid samples	Patient group (n = 30)	Control group (n = 10)
Mean age ± SD (range)	31.5 ± 6.58 (20 – 45)	34.5 ± 2.06 (32 – 37)*
Hormonal intake		
Yes	1	-
No	-	-
No data	29	10
Disease stage		
rASRM I-II	13	-
rASRM III-IV	17	-

*Unfortunately, we were able to obtain age information for only 4 of the 10 control subjects.

Table S6. Patient and control clinical characteristics from individuals recruited for urine methylhistamine measurement.

	Patient group (n = 35)	Control group (n = 25)
Mean age ± SD (range)	31.6 ± 7.9 (17 – 46)	31.3 ± 6.2 (19 – 43)
Hormonal intake		
Yes	22	7
No	13	18
Cycle phase		

Medication	17	6
Secretory	10	10
Proliferative	4	6
Menstruation	2	2
No data	2	1
Endometriosis diagnosis		
Endometriosis genitalis externa	9	-
Adenomyosis uteri	6	
Both	20	
